# Inequalities in sexual and reproductive outcomes among women aged 16–24 in England (2012–2019)

**DOI:** 10.1136/jech-2023-220835

**Published:** 2024-04-12

**Authors:** Danielle Solomon, Jo Gibbs, Fiona Burns, Hamish Mohammed, Stephanie J Migchelsen, Caroline A Sabin

**Affiliations:** 1 Institute for Global Health, University College London, London, UK; 2 National Institute for Health Research (NIHR) Health Protection Research Unit (HPRU) in Blood Borne and Sexually Transmitted Infections at UCL in Partnership with UK Health Security Agency (UKHSA), London, UK; 3 Royal Free London NHS Foundation Trust, London, UK; 4 University College London, London, UK; 5 Blood Safety, Hepatitis, Sexually Transmitted Infections (STI) and HIV Division, UK Health Security Agency, London, UK

**Keywords:** health inequalities, sexual health, reproductive health, sexually transmitted diseases

## Abstract

**Background:**

Women aged 16–24 in England have a high burden of sexual and reproductive morbidity, with particularly poor outcomes among people living in more deprived areas (including racially minoritised populations). This analysis used national data to examine the disparities within sexual and reproductive outcomes among this population and to assess whether the patterns of inequality were consistent across all outcomes.

**Methods:**

Within this ecological study, univariable and multivariable Poisson regression analyses of neighbourhood-level data from national data sets were carried out to investigate the relationships of deprivation and ethnicity with each of six dependent variables: gonorrhoea and chlamydia testing rates, gonorrhoea and chlamydia test positivity rates, and abortion and repeat abortion rates.

**Results:**

When comparing Index of Multiple Deprivation (IMD) decile 1 (most deprived) and IMD decile 10 (least deprived), chlamydia (RR 0.65) and gonorrhoea (0.79) testing rates, chlamydia (0.70) and gonorrhoea (0.34) positivity rates, abortion rates (0.45) and repeat abortion rates (0.72) were consistently lower in IMD decile 10 (least deprived). Similarly, chlamydia (RR 1.24) and gonorrhoea positivity rates (1.92) and repeat abortion rates (1.31) were higher among black women than white women. Results were similar when both ethnicity and deprivation were incorporated into multivariable analyses.

**Conclusion:**

We found similar patterns of outcome inequality across a range of sexual and reproductive outcomes, despite multiple differences in the drivers of each outcome. Our analysis suggests that there are broad structural causes of inequality across sexual and reproductive health that particularly impact the health of deprived and black populations.

**WHAT IS ALREADY KNOWN ON THIS TOPIC**

- There is a correlation between sociodemographic factors and sexual and reproductive health outcomes, with outcomes often being worse among racially minoritised people and those living in more deprived areas.

**WHAT THIS STUDY ADDS**

- Few studies have explored the consistency of patterns of inequality across sexual and reproductive outcomes.
- This analysis examines disparities in chlamydia and gonorrhoea positivity rates and repeat abortion rates, outcomes that are rarely investigated.

**HOW THIS STUDY MIGHT AFFECT RESEARCH, PRACTICE OR POLICY**

- There is a consistent pattern of outcome disparity across sexual and reproductive health among women aged 16–24.
- Policymakers and commissioners should consider the interconnected nature of sexual and reproductive health and the structural drivers of outcome inequality when attempting to tackle disparities among this population.

## Introduction

There is a large burden of sexual and reproductive morbidity in England, a burden that disproportionately affects people under the age of 25, particularly women. Rates of sexually transmitted infection (STI) diagnosis in England are highest among people within this age group, with women of this age being more likely to be diagnosed with an STI than men.^1^ Women aged 16–24 are also more likely to experience an unplanned pregnancy than older women,^2^ and this age group has the highest rate of abortion.^3^ Multiple previous studies have indicated a correlation between demographic factors and sexual and reproductive health outcomes, with outcomes routinely being worse among racially minoritised people and those living in more deprived areas.^4–7^ There has, however, been little investigation of the consistency between patterns of inequality within sexual and reproductive outcomes within this population. While sexual and reproductive health are experientially intertwined for young women in England,^8^ the fragmentation of sexual and reproductive services with commissioning, policy and delivery can make good sexual and reproductive outcomes increasingly unattainable, particularly for women from the most vulnerable populations.^9^ Understanding the similarities between patterns of inequality within a range of sexual and reproductive outcomes can aid in our understanding that cross-cut this area of health.

The aim of this analysis was to use national data to examine the disparities within sexual and reproductive outcomes among women aged 16–24 in England. In particular, we considered associations of deprivation and ethnicity with multiple health outcomes (gonorrhoea testing, gonorrhoea diagnosis, chlamydia testing, chlamydia diagnosis, abortions and repeat abortions), in order to assess whether the patterns of inequality seen within this population were consistent across sexual and reproductive outcomes, despite a wide variation in the drivers of those outcomes.

## Methods

This analysis was limited to people aged 16–24 who identified as women at the time of testing or termination of pregnancy. Data for these analyses were obtained from the two national STI surveillance systems collected by the UK Health Security Agency (the GUMCAD STI Surveillance System (GUMCAD) and the CTAD Chlamydia Surveillance System (CTAD)) and the Department of Health and Social Care (DHSC) Abortion Dataset. Detailed protocols for all three of these data sets have been published elsewhere.^3 10 11^ Three aggregate data sets were generated, which included information on: (1) all gonorrhoea tests and diagnoses among women aged 16–24 in England between 2012 and 2019, (2) all chlamydia tests and diagnoses among the same population and (3) all abortions among this population, and whether the person undergoing the abortion had undergone one or more previous abortions (ie, whether the abortion was a repeat abortion). For each test and abortion, we had a record of the patient’s residential postcode and ethnicity (using standardised ethnic categories defined by the UK’s Office for National Statistics).

This was an ecological study that examined all outcomes at the neighbourhood (rather than individual) level. The unit of analysis was the lower layer super output area (LSOA), a small geographical unit with a mean population of 1500 people. The population of women aged 16–24 living in each of the 33 755 LSOAs in England from 2012 to 2019 was calculated using population estimates provided by the Office for National Statistics (ONS).^12^ The level of deprivation within an LSOA was defined as the decile into which the LSOA fell within the 2015 Index of Multiple Deprivation (IMD), a validated tool that is routinely used to quantify deprivation at the neighbourhood level in England.^13^

To ensure a large enough sample size within each ethnic group, ethnicity (as determined by the self-identification provided at the time of testing) was classified as white (White British, White Irish, White Other), black (black Caribbean, black African or black Other) or Asian (Bangladeshi, Chinese, Indian, Pakistani or Asian Other); people who reported mixed ethnicity were placed into the relevant minoritised subgroup (mixed white and Asian was incorporated into Asian, while both White and Black Caribbean and White and Black African were incorporated into black). For analyses involving ethnicity, we restricted the data set to those who identified their ethnicity as Asian, Black or White, and excluded those with other or unknown ethnicities. No data other than ethnicity were missing from the dataset.

Poisson regression analyses were carried out to investigate the relationship between deprivation and each of six dependent variables: gonorrhoea and chlamydia testing rates (per 1000 population), gonorrhoea and chlamydia test positivity rates (per 1000 tests) and abortion (per 1000 population) and repeat abortions (per 1000 abortions) rates.

We were unable to examine the relationship between testing and abortion rates and ethnicity, as this would require a measure of LSOA population size stratified by gender, age and ethnicity (as the denominator for these outcomes is a population measure), and these data are not provided by the ONS. We, therefore, carried out Poisson regression analyses examining the relationships between ethnicity and three of the outcomes: gonorrhoea test positivity rate, chlamydia test positivity rate and repeat abortion rate. To examine the independent associations of deprivation and ethnicity with the outcomes within this study, we also performed multivariable analyses of the associations of both deprivation and ethnicity with these three outcomes.

All analyses were carried out using STATA V.17 (Stata 2021. Stata Statistical Software: Release V.17. College Station, Texas: Stata).

This analysis received ethical approval from the UCL Research Ethics Committee (approval ID number: 19369/002).

## Results

### Gonorrhoea

**Figure 1:**
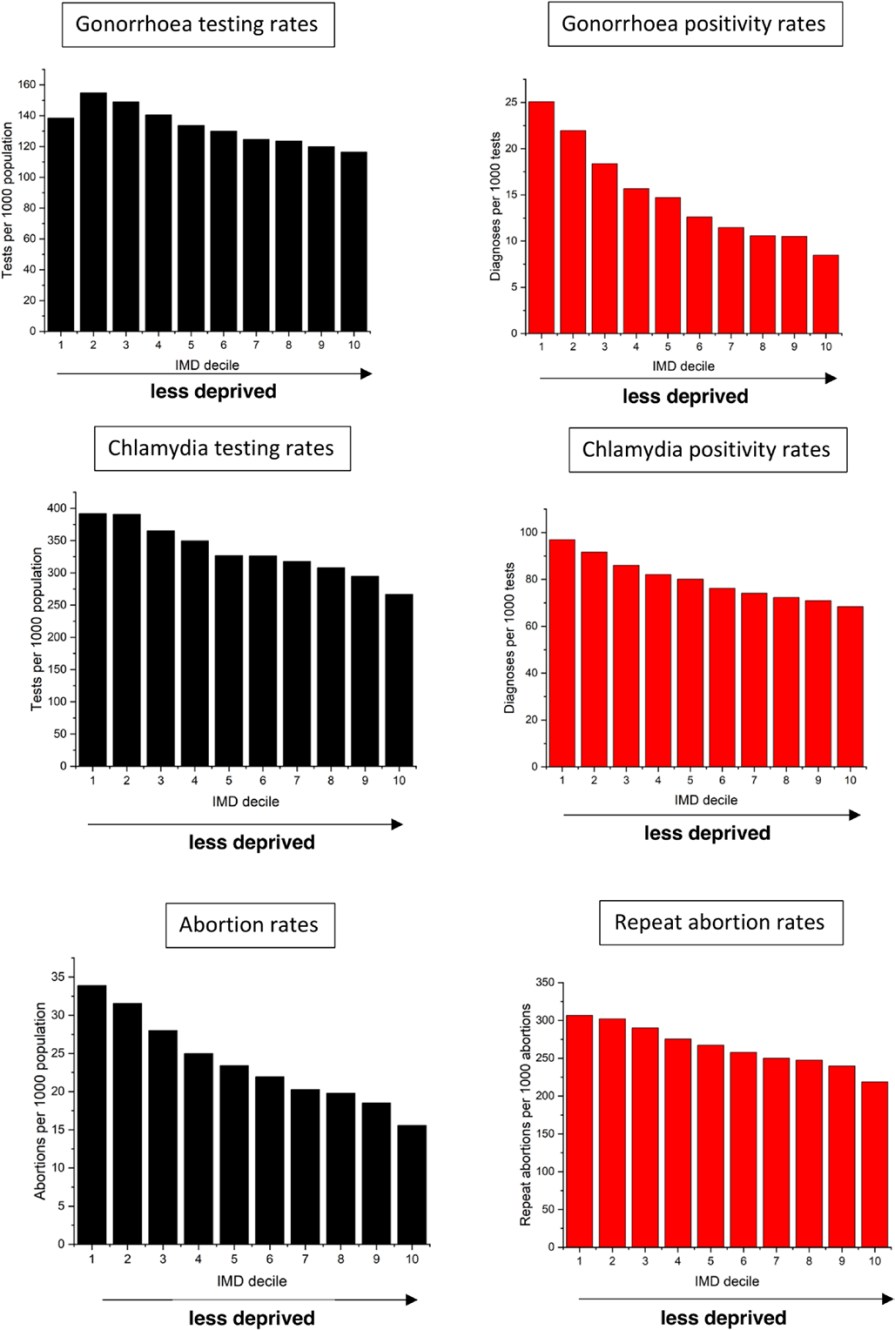
Relationships between sexual and reproductive outcomes and deprivation among women aged 16–24. IMD, Index of Multiple Deprivation.

**Figure 2:**
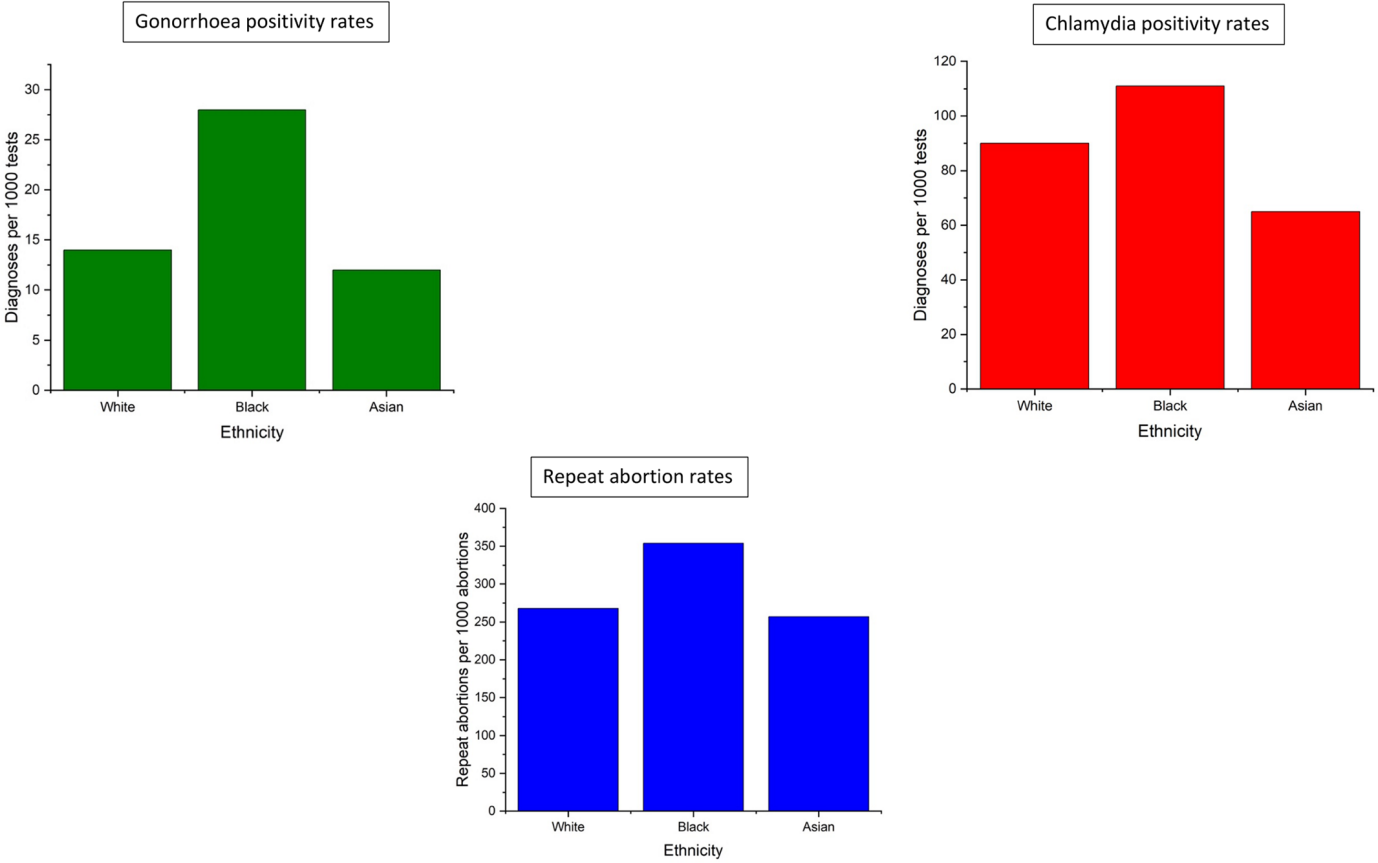
Relationships between sexual and reproductive outcomes and ethnicity among women aged 16–24.

From 2012–2019, 3 220 976 gonorrhoea tests were recorded within the target population, 51 308 (1.59%) of which were positive. There was an inverse relationship between IMD decile and both outcomes, with greater testing and a higher test positivity rate in women in more deprived areas (figure 1). Similarly, women of black ethnicity had significantly higher rates of gonorrhoea positivity than women of White or Asian ethnicity (figure 2).

#### Deprivation

In unadjusted regression analyses, the rate ratio (RR) for the relationship between the testing rate in deciles 1 and 10 was 0.87 (95% CI 0.86 to 0.88), indicating that the testing rate in the least deprived decile of the population was about 90% of the testing rate in the most deprived decile. Similarly, the RR for the relationship between the positivity rate in deciles 1 and 10 was 0.34 (0.33 to 0.36), indicating that the positivity rate in the least deprived areas was about a third that seen in the most deprived areas (table 1).

**Table 1:** Relationship between deprivation, ethnicity and sexual health outcomes within this study

		Gonorrhoea testing rate (tests/1000 population)	Gonorrhoea positivity rate (diagnoses/1000 tests)		Chlamydia testing rate (tests/1000 population)	Chlamydia positivity rate (diagnoses/1000 tests)	
IMD (2015)		Rate ratio (95% CI) univariable	Rate ratio (95% CI) univariable	Rate ratio (95% CI) multivariable	Rate ratio (95% CI) univariable	Rate ratio (95% CI) univariable	Rate ratio (95% CI) multivariable
Most deprived	1	Reference category	Reference category	Reference category	Reference category	Reference category	Reference category
	2	1.11 (1.10 to 1.11)	0.86 (0.84 to 0.89)	0.84 (0.82 to 0.87)	0.98 (0.98 to 0.99)	0.94 (0.93 to 0.94)	0.92 (0.91 to 0.93)
	3	1.03 (1.03 to 1.03)	0.73 (0.71,0.75)	0.73 (0.71 to 0.76)	0.93 (0.93 to 0.93)	0.89 (0.88 to 0.89)	0.87 (0.87 to 0.88)
	4	0.97 (0.97 to 0.98)	0.62 (0.60 to 0.64)	0.64 (0.62 to 0.66)	0.88 (0.88 to 0.88)	0.85 (0.84 to 0.85)	0.84 (0.83 to 0.84)
	5	0.91 (0.91 to 0.92)	0.58 (0.56 to 0.60)	0.60 (0.58 to 0.63)	0.81 (0.80 to 0.81)	0.82 (0.81 to 0.82)	0.80 (0.80 to 0.81)
	6	0.85 (0.85 to 0.85)	0.49 (0.47 to 0.51)	0.52 (0.50 to 0.54)	0.77 (0.77 to 0.77)	0.78 (0.78 to 0.79)	0.77 (0.77 to 0.78)
	7	0.85 (0.85 to 0.86)	0.44 (0.42 to 0.46)	0.47 (0.45 to 0.49)	0.77 (0.77 to 0.78)	0.76 (0.75 to 0.77)	0.75 (0.74 to 0.76)
	8	0.84 (0.84 to 0.84)	0.43 (0.41 to 0.45)	0.46 (0.44 to 0.48)	0.77 (0.77 to 0.77)	0.75 (0.75 to 0.76)	0.74 (0.74 to 0.75)
	9	0.82 (0.82 to 0.83)	0.42 (0.40 to 0.44)	0.45 (0.43 to 0.47)	0.74 (0.74 to 0.74)	0.74 (0.73 to 0.74)	0.73 (0.72 to 0.73)
	10	0.79 (0.82 to 0.83)	0.34 (0.33 to 0.36)	0.37 (0.36 to 0.39)	0.65 (0.65 to 0.65)	0.70 (0.70 to 0.71)	0.69 (0.68 to 0.70)

Ethnicity	White	Reference category	Reference category	Reference category	Reference category	Reference category	Reference category
	Black		1.92 (1.88 to 1.97)	1.63 (1.59 to 1.66)		1.24 (1.23 to 1.25)	1.16 (1.15 to 1.17)
	Asian		0.81 (0.77 to 0.85)	0.75 (0.71 to 0.79)		0.76 (0.74 to 0.77)	0.73 (0.72 to 0.75)

IMD, Index of Multiple Deprivation.

#### Ethnicity

Of the tests and diagnoses included in these analyses, 269 724 tests (8.4%) and 4217 diagnoses (8.2%) were among women who reported an ethnicity other than White, Black or Asian (or no ethnicity) and were subsequently excluded from ethnicity analyses (table 2). Among the remaining tests and diagnoses, the test positivity rate also varied significantly by ethnic group (p<0.001). When compared with the test positivity rate among women who identified as white, tests performed on women who identified as black were almost two times as likely to be positive (RR 1.92 (95% CI 1.88 to 1.97)). The test positivity rate among women who identified as Asian was lower than that among women who identified as white (0.81 (0.77 to 0.85)).

**Table 2:** Overall characteristics of women whose tests and diagnoses were included in this analysis

	Gonorrhoea tests		Gonorrhoea diagnoses		Chlamydia tests		Chlamydia diagnoses	
	N	%	N	%	N	%	N	%
**Total**	3 220 976	100	51 308	100	8 103 547	100	664 040	100
IMD (2015)								
1 (most deprived)	395 176	12.3	9908	19.3	1 097 103	13.5	106 496	16.0
2	444 622	13.8	9618	18.7	1 095 938	13.5	99 645	15.0
3	416 148	12.9	7622	14.9	1 042 653	12.9	89 642	13.5
4	377 717	11.7	5901	11.5	949 156	11.7	77 857	11.7
5	320 608	10.0	4655	9.1	786 691	9.7	62 355	9.4
6	281 422	8.7	3482	6.8	707 830	8.7	53 881	8.1
7	267 518	8.3	2954	5.8	672 837	8.3	49 456	7.4
8	253 766	7.9	2733	5.3	645 367	8.0	47 271	7.1
9	239 040	7.4	2501	4.9	597 192	7.4	42 623	6.4
10	224 939	7.0	1934	3.8	508 780	6.3	34 814	5.2
Ethnicity								
White	2 439 262	75.7	35 001	68.2	3 760 109	46.4	338 328	50.9
Black	384 291	11.9	10 611	20.7	504 065	6.2	56 226	8.5
Asian	127 699	3.9	1479	2.9	191 940	2.4	13 070	1.9
Any other ethnicity	39 346	1.2	677	1.3	53 130	0.7	4633	0.7
Not known/not stated	178 243	5.5	2467	4.8	3 522 116	43.5	244 922	36.9

IMD, Index of Multiple Deprivation.

#### Multivariable analyses

Within our data set, we found a significant correlation between ethnicity and deprivation (p<0.001), with tests among women of White British ethnicity being more likely to be linked to a less deprived LSOA than tests among racially minoritised women. Controlling the deprivation analyses for ethnicity (and controlling the ethnicity analyses for deprivation) did not have a large impact on the estimates from the model for gonorrhoea test positivity rates. In particular, the adjusted RR (aRR) for IMD decile 10 was 0.37 (0.35 to 0.39) when compared with IMD decile 1, with aRRs for black 1.62 (1.59 to 1.66) and Asian 0.75 (0.71 to 0.79) women remaining similar to unadjusted estimates when compared with White women (table 1).

### Chlamydia

During the analysis period, 8 103 547 chlamydia tests were recorded within the target population, 664 040 (8.2%) of which were positive. As with gonorrhoea, there was an inverse relationship between chlamydia testing and positivity and IMD (figure 1), and chlamydia test positivity was highest among women of black ethnicity.

#### Deprivation

On regression analysis, the rate RR for the relationship between the testing rate in deciles 1 and 10 was 0.65 (0.65 to 0.65), and the RR for the positivity rate was 0.70 (0.70 to 0.71) (table 1).

#### Ethnicity

Of the tests and diagnoses included in these analyses, 3 647 433 tests (45.0%) and 256 416 diagnoses (38.6%) were among women who reported an ethnicity other than White, Black or Asian (or no ethnicity) and were subsequently excluded from ethnicity analyses (table 2). Positivity rate once again varied by ethnic group (p<0.001) (figure 2). When compared with tests among women who identified as white (90.0/1000 tests), tests performed on women who identified as black were more likely to be positive (RR 1.22 (1.21 to 1.23), while tests among women who identified as Asian less likely to be positive (RR 0.73 (0.71 to 0.74)) (table 1).

#### Multivariable analyses

As with gonorrhoea, chlamydia tests among women of White British ethnicity were more likely to be linked to a less deprived LSOA than tests among racially minoritised women (p<0.001). Once again, controlling the deprivation analysis for ethnicity (and vice versa) did not have a large impact on the overall relationship between deprivation, ethnicity and chlamydia positivity rates. The aRR for IMD decile 10 was 0.69 (0.68 to 0.70) when compared with IMD decile 1, with ARRs for black 1.16 (1.15 to 1.17) and Asian 0.73 (0.72 to 0.75) women remaining similar to unadjusted estimates when compared with White women (table 1).

### Abortion

During the analysis period, 585 641 abortions were recorded within the analysis population, 160 971 (27.5%) of which were repeat abortions. As with the STIs, there was an inverse relationship between both abortion rates and repeat abortion rates, and deprivation (figure 1) and abortions among Black women were more likely to repeat abortions than those among White or Asian women (figure 2).

#### Deprivation

In unadjusted regression analyses, the RR for the relationship between the abortion rate in deciles 1 and 10 was 0.45 (0.44 to 0.46) and the RR for the relationship between the repeat abortion rate in deciles 1 and 10 was 0.72 (0.70 to 0.74) (table 3).

**Table 3:** Relationship between deprivation, ethnicity and reproductive health outcomes within this study

		Abortion rate (abortions/1000 population	Repeat abortion rate (repeat abortions/1000 abortions)	
IMD(2015)		Rate ratio (95% CI) univariable	Rate ratio (95% CI) univariable	Rate ratio (95% CI) multivariable
Most deprived	1	Reference category	Reference category	Reference category
	2	0.93 (0.92, 0.94)	0.98 (0.97, 1.00)	0.97 (0.96, 0.99)
	3	0.81 (0.81, 0.82)	0.94 (0.92, 0.95)	0.94 (0.92, 0.95)
	4	0.73 (0.72, 0.74)	0.89 (0.88, 0.91)	0.90 (0.88, 0.91)
	5	0.67 (0.66, 0.68)	0.86 (0.85, 0.88)	0.87 (0.85, 0.89)
	6	0.62 (0.61, 0.63)	0.84 (0.82, 0.86)	0.85 (0.83, 0.87)
	7	0.59 (0.58, 0.59)	0.82 (0.80, 0.84)	0.83 (0.81, 0.85)
	8	0.58 (0.57, 0.58)	0.80 (0.78, 0.82)	0.81 (0.79, 0.83)
	9	0.54 (0.53, 0.54)	0.78 (0.76, 0.80)	0.79 (0.77, 0.81)
	10	0.45 (0.44, 0.46)	0.72 (0.70, 0.74)	0.73 (0.71, 0.75)
Ethnicity				
	White	Reference category	Reference category	Reference category
	Black		1.31 (1.29,1.33)	1.25 (1.24, 1.27)
	Asian		0.95 (0.94, 0.97)	0.93 (0.91, 0.95)

IMD, Index of Multiple Deprivation.

#### Ethnicity

Of the abortions included in these analyses, 25 350 abortions (4.33%) and 6070 repeat abortions (3.77%) were undergone by women who reported an ethnicity other than white, black, Asian (or no ethnicity) (table 4) and were, therefore, excluded from the ethnicity analyses. As with the STI positivity analyses, repeat abortion rate correlated with ethnic group (p<0.001) (figure 2). Abortions undergone by women who identified as black were more likely to be repeat abortions than those undergone by women who identified as white (RR 1.31 (1.39 to 1.33)), while abortions among women who identified as Asian (255.9/1000 abortions) were less likely to be repeat abortions (RR 0.95 (0.94 to 0.97)).

**Table 4:** Overall characteristics of women whose abortions were included in this analysis

	Abortions		Repeat abortions	
	N	%	N	%
Total	585 641	100	160 971	100
IMD (2015)				
1 (most deprived)	77 584	13.2	22 852	14.2
2	75 912	13.0	22 538	14.0
3	70 594	12.1	19 857	12.3
4	61 823	10.5	17 035	10.6
5	57 023	9.73	15 640	9.72
6	52 961	9.04	14 058	8.73
7	49 868	8.52	12 943	8.04
8	48 656	8.31	12 536	7.79
9	46 984	8.02	12 314	7.65
10	44 236	7.55	11 198	6.96
Ethnicity				
White	453 024	77.34	121 454	75.41
Black	58 237	9.95	20 429	12.69
Asian	42 048	7.18	10 758	6.68
Any other ethnicity	6491	1.11	1631	1.01
Not known/not stated	18 859	3.22	4439	2.76

IMD, Index of Multiple Deprivation.

#### Multivariable analysis

Abortions among women of White British ethnicity were significantly more likely to be linked to a less deprived LSOA than those among racially minoritised women (p<0.001). Once again, controlling the deprivation analysis for ethnicity (and vice versa) did not have a large impact on the overall relationship between deprivation, ethnicity and repeat abortion rates. The aRR for IMD decile 10 was 0.73 (0.71 to 0.75) when compared with IMD decile 1, with aRRs for black 1.25 (1.24 to 1.27) and Asian 0.93 (0.91 to 0.95) women remaining similar to unadjusted estimates when compared with white women (table 3).

## Discussion

The results of our analyses indicate a pattern of inequality within sexual and reproductive health among young women in England. Although the gradient of inequality differed, black women and those living in the most deprived areas were consistently found to have worse sexual and reproductive outcomes than their white and less deprived peers. Many of the outcomes that we investigated are underexamined within the published literature, in particular, gonorrhoea test positivity, chlamydia test positivity and repeat abortion. The fact that we have seen similar patterns of disparity across these outcomes, each of which has a range of different drivers, suggests that wider structural inequalities are having a broad impact on the sexual and reproductive health of this population.

With regards to the two STIs examined within this analysis, the fact that testing rates for gonorrhoea and chlamydia are higher in deprived LSOAs and that these tests are more likely to be positively appears to indicate an increased demand for services in more deprived areas that is concurrent with an increased risk of infection. Although we were unable to examine differences in testing rates by ethnicity, the consistently increased likelihood of a positive test among black women when compared with white women may also be evidence of a higher prevalence among this ethnic group.

Trends within chlamydia testing rates and test positivity rates among young women in England are likely to be affected by the National Chlamydia Screening Programme (NCSP), a national programme of opportunistic chlamydia screening aimed at sexually active women under the age of 25.^14^ It is possible that the strategic direction of the NCSP could affect our interpretation of the results of our analysis—for example, higher testing and positivity rates in historically underserved groups could be a result of deliberately targeted screening, rather than higher prevalence. However, the fact that the patterns of disparity within chlamydia testing and diagnosis mirror those seen when looking at gonorrhoea (an STI that is not linked to a screening programme) make these disparities less likely to be driven by screening, although the activity of the NCSP may explain the fact that the disparities that correlate with deprivation and ethnicity are much smaller for chlamydia than they are for gonorrhoea.

When examining abortion rates, the conclusions drawn from the disparities seen within our analyses are particularly complex. Unlike the STI analyses, which focused on an outcome that is inherently undesirable (chlamydia and gonorrhoea infection), there are logistical, structural and ethical barriers to deciding that a certain incidence of abortion indicates a negative outcome.^15^ Although higher abortion rates among certain groups may indicate a need for prevention of unplanned pregnancy, this may also indicate a need for abortion access among the groups who have a lower rate of abortion. Drivers of repeat abortion are even more complex. Numerous studies have found that (contrary to popular opinion^16^) women who have two or more abortions do not have a lower likelihood of contraceptive use than those who have a single abortion. Instead, type of contraceptive used (particularly oral contraceptives,^17^ availability and access to preferred contraceptive method,^18^ a history of relationship violence^17 19^ and greater number of sexual partners^16^ have all been found to correlate with repeat abortion. It is, therefore, likely that the disparity in abortion rates is the result of a combination of clinical, educational and structural needs within more deprived populations, and populations of certain ethnicities.

Our multivariable analyses indicated that the distribution of ethnic groups does not appear to be a significant contributor to the negative correlation between IMD and positivity/abortion rates, and similarly, controlling the ethnicity analyses for deprivation did not have a large impact on the relationship between positivity/abortion rate and ethnicity. While these two relationships mirror each other, it would appear that they have separate causes, indicating that both relationships would benefit from further, mixed-methods, investigation at a more granular level.

### Strengths and weaknesses

The data sets that we created for these analyses included data at the LSOA level. As GUMCAD, CTAD and abortion data were aggregated for the purposes of this analysis, there was no way to disaggregate first time versus repeat testing. This, combined with the fact that the data set is naturally biased towards people who present for testing/abortion care, makes it very difficult to draw any conclusions from these analyses about any absolute metrics such as population incidence or prevalence. It is also difficult to draw conclusions about individual associations from these analyses, without falling victim to the ecological fallacy (making erroneous assumptions about individual outcomes using aggregated data^20^). Further research on this topic should, therefore, include analyses of individual-level data, which may shed light on the drivers of some of the disparities seen within this analysis.

Another limitation is the use of the IMD deciles that were calculated in 2015 for this analysis. The 2015 IMD calculations used data that were largely collected between 2012 and 2015 (while the 2019 IMD calculations predominantly used data collected from 2015 to 2019), which means that the IMD decile used for the outcomes within this analysis that were recorded after 2015 may be out of date. The lack of data outlining population size stratified by ethnicity at the LSOA level was also a limitation, as we were unable to examine the relationship between ethnicity and testing or abortion rates. In addition, the chlamydia data set within this analysis is missing a large amount of ethnicity data (nearly half of tests within this data set were performed on a person whose ethnicity was not recorded). There is a concern that this missing data may be systematic, that is, people of certain ethnicities may be more likely to report their ethnicity than others. However, our missing ethnicity data were evenly split across IMD deciles, which makes a systematic loss of ethnicity data less likely. In addition, the similarities between the trends seen within the chlamydia data set and the gonorrhoea and abortion data sets support the inclusion of the ethnicity-related analyses of chlamydia positivity rates within this analysis.

Despite these challenges, this analysis has a number of strengths. The use of a very large sample from three national surveillance data sets allowed us to draw robust conclusions about patterns of inequality within sexual and reproductive health. In particular, very few analyses of abortion trends have used nationally collected data to investigate the patterns of abortion among various demographics, and data on the distribution of repeat abortions are particularly scarce.

## Conclusions

Overall, the fact that the pattern of disparities seen within our analyses persists across a range of outcomes suggests that there are structural causes of inequality that have an impact on both sexual health and reproductive health. When attempting to address these inequalities, it is, therefore, important to recognise the interconnected nature of sexual and reproductive health, particularly among women under the age of 25.^21^ We believe that further investigation into the structural connections between sexual and reproductive health would help improve our understanding of the overlapping inequalities within this area, and we would recommend a more holistic approach to the design, commissioning and provision of sexual and reproductive health services, to ensure that the fragmentation of services does not act as an additional structural barrier to healthcare access among the most vulnerable populations.

## Data Availability

Data may be obtained from a third party and are not publicly available. Data used in this analysis are held by the Department of Health and Social Care and the UK Health Security Agency, and as such are not publicly available.

## References

[1] Mitchell H , Allen H , Sonubi T , et al . Sexually transmitted infections and screening for chlamydia in England, 2019 Public Health England, 2020. Available: https://www.gov.uk/government/statistics/sexually-transmitted-infections-stis-annual-data-tables

[2] Wellings K , Jones KG , Mercer CH , et al . The prevalence of unplanned pregnancy and associated factors in Britain: findings from the third national survey of sexual attitudes and lifestyles (Natsal-3). Lancet 2013;382:1807–16. 10.1016/S0140-6736(13)62071-1 24286786 PMC3898922

[3] Department of Health and Social Care . Abortion statistics, England and Wales. 2019.

[4] Sales JM , Smearman EL , Swartzendruber A , et al . Socioeconomic-related risk and sexually transmitted infection among African-American adolescent females. J Adolesc Health 2014;55:698–704. 10.1016/j.jadohealth.2014.05.005 24974317 PMC4209307

[5] Newbern EC , Miller WC , Schoenbach VJ , et al . Family socioeconomic status and self-reported sexually transmitted diseases among black and white American adolescents. Sex Transm Dis 2004;31:533–41. 10.1097/01.olq.0000137898.17919.35 15480114

[6] Sonnenberg P , Clifton S , Beddows S , et al . Prevalence, risk factors, and uptake of interventions for sexually transmitted infections in Britain: findings from the national surveys of sexual attitudes and lifestyles (Natsal). The Lancet 2013;382:1795–806. 10.1016/S0140-6736(13)61947-9 PMC3899025 24286785

[7] Mooney J , Yau R , Moiz H , et al . Associations between socioeconomic deprivation and pharmaceutical prescribing in primary care in England. Postgrad Med J 2022;98:193–8. 10.1136/postgradmedj-2020-138944 33310893

[8] Office for Health Improvement and Disparities . Integrated sexual health service specification. 2023.

[9] Local Government Association . Derby and Derbyshire: Tackling the fragmentation of the sexual health system, 2023. Available: https://www.local.gov.uk/case-studies/derby-and-derbyshire-tackling-fragmentation-sexual-health-system

[10] England PH . GUMCAD STI Surveillance System: Data specification and Technical Guidance, 2020. Available: https://www.gov.uk/guidance/gumcad-sti-surveillance-system

[11] GOV.UK . CTAD Chlamydia Surveillance System, 2023. Available: https://www.gov.uk/guidance/ctad-chlamydia-surveillance-system

[12] Office for National Statistics . Population estimates, Available: https://www.ons.gov.uk/peoplepopulationandcommunity/populationandmigration/populationestimates

[13] Mclennan AD , Noble S , Noble M , et al . English Indices of Deprivation 2019 - Technical report, 2019. Available: https://assets.publishing.service.gov.uk/government/uploads/system/uploads/attachment_data/file/833951/IoD2019_Technical_Report.pdf

[14] GOV.UK . NCSP: programme overview, Available: https://www.gov.uk/government/publications/ncsp-programme-overview/ncsp-programme-overview

[15] Public Health England . What do women say? reproductive health is a public health issue. 2017.

[16] Howe B , Kaplan HR , English C . Repeat abortions: blaming the victims. Am J Public Health 1979;69:1242–6. 10.2105/ajph.69.12.1242 574365 PMC1619322

[17] Fisher WA . Characteristics of women undergoing repeat induced abortion. Canadian Medical Association Journal 2005;172:637–41. 10.1503/cmaj.1040341 15738488 PMC550633

[18] Rowlands S . More than one abortion. J Fam Plann Reprod Health Care 2007;33:155–8. 10.1783/147118907781005047 17609073

[19] Stone N , Ingham R . Who presents more than once? repeat abortion among women in Britain. J Fam Plann Reprod Health Care 2011;37:209–15. 10.1136/jfprhc-2011-0063 21724621

[20] Portnov BA , Dubnov J , Barchana M . On ecological fallacy, assessment errors stemming from misguided variable selection, and the effect of aggregation on the outcome of Epidemiological study. J Expo Sci Environ Epidemiol 2007;17:106–21. 10.1038/sj.jes.7500533 17033679

[21] World Health Organisation . Sexual health and its linkages to reproductive health: an operational approach. 2017.

